# Skin Carotenoid Index in a large Japanese population sample

**DOI:** 10.1038/s41598-019-45751-6

**Published:** 2019-06-27

**Authors:** Akira Obana, Yuko Gohto, Werner Gellermann, Igor V. Ermakov, Hiroyuki Sasano, Takahiko Seto, Paul S. Bernstein

**Affiliations:** 1 Department of Ophthalmology, Seirei Hamamatsu General Hospital, 1-12-12 Sumiyoshi, Naka-ku, Hamamatsu City, Shizuoka 430-8558 Japan; 2 Department of Medical Spectroscopy, Institute for Medical Photonics Research, Preeminent Medical Photonics Education & Research Center, Hamamatsu University School of Medicine, Hamamatsu, Shizuoka Japan; 3 Longevity Link Corporation, Salt Lake City, UT United States of America; 4 Department of Ophthalmology and Visual Sciences, Moran Eye Center, University of Utah School of Medicine, Salt Lake City, Utah United States of America

**Keywords:** Diagnostic markers, Lasers, LEDs and light sources, Nutrition

## Abstract

Carotenoids are anti-oxidative agents. Human skin and eyes contain specific carotenoid species known to prevent various pathologies caused by oxidative stress. We quantified skin and eye carotenoid levels and investigated their potential correlation in a population including 985 Japanese patients and staff members of an ophthalmology clinic (577 men, 408 women, mean age of 69.7 ± 13.6 [SD]). Skin carotenoid (SC) and macular pigment (MP) levels were measured with reflection spectroscopy and autofluorescence imaging methods, respectively. The mean SC index was 343.1 ± 142.1 (SD). SC indices for women were higher than for men (382 vs 315, p < 0.001). Smokers and overweight subjects (BMI ≥ 25) had lower SC indices. Subjects taking lutein supplements had higher SC indices than non-supplementing subjects (415 vs 325, p < 0.001). SC and MP indices were significantly correlated. The obtained data set can be used for reference purposes by Japanese subjects and researchers interested in tissue responses to diets high in carotenoids and lutein supplementation.

## Introduction

Carotenoids are organic pigments produced by plants and algae. Their molecular structures were first described for beta-carotene and lycopene by Nobel laureate P Karrer in 1930^1^. The structures are composed of eight isoprene molecules and a total of 40 carbon atoms. The carbon atoms are arranged in a chain like fashion with alternating carbon single and double bonds. The number of double bonds varies between 9 and 11 depending on the particular carotenoid species. Karrer *et al*. found that beta-carotene serves as provitamin in the production of vitamin A. Besides their current most common use for color, odor, and taste manipulations, carotenoids also have important functions as anti-inflammatory and anticancer agents^2^. Their anti-oxidative function has been explained as resulting from quenching of singlet oxygen and the scavenging of free radicals by the conjugated carbon chain^3^. Among the more than 750 carotenoids existing in nature, humans take up about 30 carotenoids with their diet. Once deposited into various target tissues, the carotenoids are thought to provide protection against oxidative stress, which can lead to age-related pathologies as well as various types of cancers^4–6^.

Carotenoids found in human skin include lycopene, alpha-. beta-, gamma-, and delta-carotene, beta-cryptoxanthin, lutein, and zeaxanthin. They are present throughout the epidermis, dermis, and also the subcutaneous fat^4,7,8^. These carotenoids protect the skin against sunlight induced oxidation effects. Ultraviolet (UV) light has been investigated as the main generator for oxidants, while blue wavelengths of visible light were found to also produce free radicals in this tissue^9^. Lutein and zeaxanthin, which both absorb blue light, have been reported to reduce lipid peroxidation and to increase moisture levels in the skin^10^. So far, skin carotenoid levels have been investigated mainly in Caucasians, while studies in Asian populations are rather limited^11^.

The healthy human eye accumulates the three carotenoids in the retina: lutein, zeaxanthin, and *meso*-zeaxanthin^12,13^. Highest levels are typically located in the central region of the retina as macular pigment (MP) and are located anteriorly to the photoreceptors responsible for color vision. Lutein and zeaxanthin accumulate in the retina in a highly selective uptake process controlled by proteins that specifically bind only these two species^14,15^. *meso*-Zeaxanthin is produced from lutein by retinal pigment epithelium-specific protein 65 kDa (RPE65) in the retinal pigment epithelial cells^16^. The MP absorbs blue light, improves contrast sensitivity, reduces glare^17,18^, and prevents oxidative damage to photoreceptor cells and retinal pigment epithelial cells via photochemical reactions^19^. Previous reports showed low to moderate correlation coefficients between skin carotenoid (SC) levels and MP optical density (MPOD) levels (r = 0.34 in newborn infants, 0.42 in children, 0.663 in ophthalmology patients)^20–22^.

Low intake of lutein and zeaxanthin-rich foods, low concentrations of serum lutein and zeaxanthin, and low levels of MP are all risk factors for developing age-related macular degeneration (AMD)^6,23–25^. Prospective studies clarified the efficiency of eye supplements, containing lutein and zeaxanthin along with other antioxidant vitamins, in reducing the disease progression to advanced AMD^26^. The beneficial effects of these supplements are difficult to perceive on an individual basis, and patients often stop supplementation due to missing feedback on their MP status. If a quantitative and easily applicable diagnostic method that could visualize dietary and/or supplementation effects were to exist, it could potentially result in fewer AMD cases. A direct measurement of MP levels would be most desirable for this purpose, but the two currently existing diagnostic methods, which are based on heterochromatic flicker photometry and autofluorescence imaging methods, respectively, have inherent limitations. Flicker photometry has a relatively long examination time and is difficult to use with the elderly; autofluorescence imaging requires mydriasis in combination with high light intensities for accurate MP measurements, and it is strongly susceptible to lens opacification occurring with increasing subject age. In contrast, indirect assessment via SC level measurements could provide a non-invasive rapid screening alternative, which can be carried out with resonance Raman spectroscopy (RRS) or reflection spectroscopy (RS) methods^7,27–30^.

RRS has already been used for almost two decades in the nutritional supplement industry. Since RRS measures Raman signals of carotenoid vibrations, it has high specificity and precision. However, it requires a narrow-band excitation source, a high-resolution spectrometer, and, due to the relatively weak Raman signals, it also requires highly sensitive detection schemes. In comparison, RS can take advantage of the relatively strong light signals reflected from the skin, and the instrumentation can be configured with relatively simple light sources, filters and/or low-resolution spectrographs. RS has a slightly reduced specificity and precision compared to RRS, but RS is less complex than RRS, less expensive, and has the potential for widespread use also outside the nutritional supplement industry.

In the present study, we evaluated SC levels in more detail and in a larger sample size compared to our previous study^11^. Also, we investigated the correlation with MP levels. The obtained SC indices can serve as an initial national database in Japan.

## Results

### Subject characteristics

**Figure 1:**
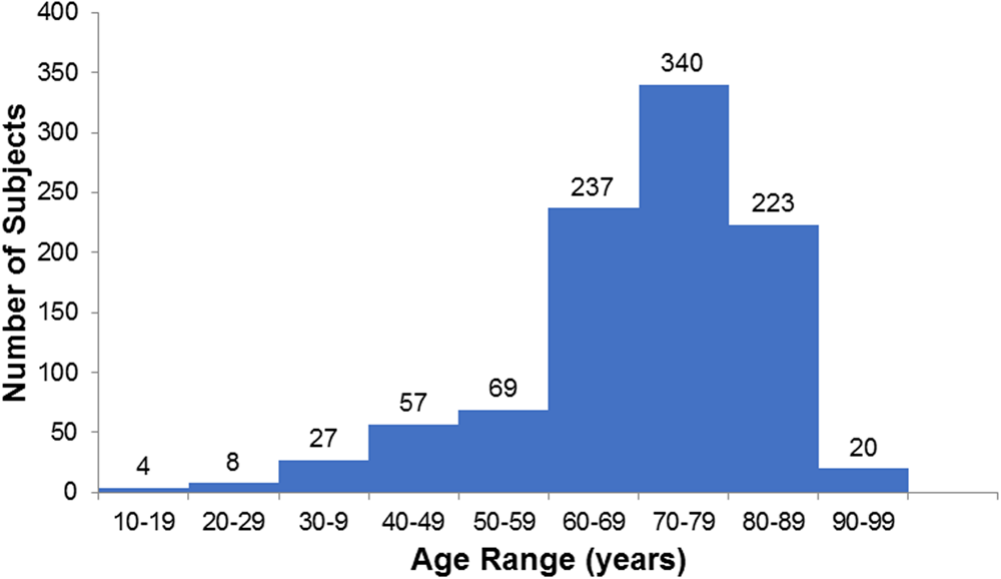
Histogram of subject age. The mean was 69.7 ± 13.8 (SD) years old.

Table 1 shows the demographic data for all subjects. Since the majority of the subjects were patients of an ophthalmology clinic, the subject age is skewed to higher ages (Fig. 1). Significant differences among patients existed for age, tobacco smoking history, body mass index (BMI), lutein supplementation history, history of diabetes, and the various types of macular disorders. Men were older, had a more frequent tobacco smoking habit, higher BMI, and a higher rate of diabetes than women. Lutein supplementation was more frequent in women; AMD occurrence was more frequent in men.

**Table 1:** Demographic data of all subjects.

	Total (985)	Male (577)	Female (408)	*P*
Age range (years)	16–97	18–93	16–97	
Mean age (SD)	69.7 (13.6)	70.7 (12.4)	68.3 (15.1)	**0.008 (Welch’s t-test)**
Tobacco*	N499, P316, C122, Unknown 3	N160, P302, C115, Unknown 0	N362, P28, C15, Unknown 3	**<0.001 (chi-square)**
BMI range	12.5–39.6	15.2–39.6	12.5–34.5	
Mean BMI (SD)	22.7 (3.5)	23.2 (3.4)	22.1 (3.6)	**<0.001 (t-test)**
Lutein supplement	N737, Y190, Unknown 4	N477, Y100, Unknown 0	N308, Y95, Unknown 5	**0.002 (chi-square)**
Diabetes	N756, Y175	N457, Y120	N344, Y64	**0.025 (chi-square)**
NDR	759	69	38	0.989 (chi-square)
SDR	101	8	4	
PPDR	12	19	9	
PDR	18	24	13	
Macular disorder				
No disorder	190	114	141	**<0.001 (chi-square)**
AMD	435	320	122	
Others	262	143	145	

SD, standard deviation; *N, non-smoker, P, past smoker, C, current smoker; BMI, body mass index; NDR, no diabetic retinopathy; SDR, simple diabetic retinopathy, PPDR, pre-proliferative diabetic retinopathy, PDR, proliferative diabetic retinopathy; AMD, age-related macular degeneration.

### Skin carotenoid (SC) indices

**Figure 2:**
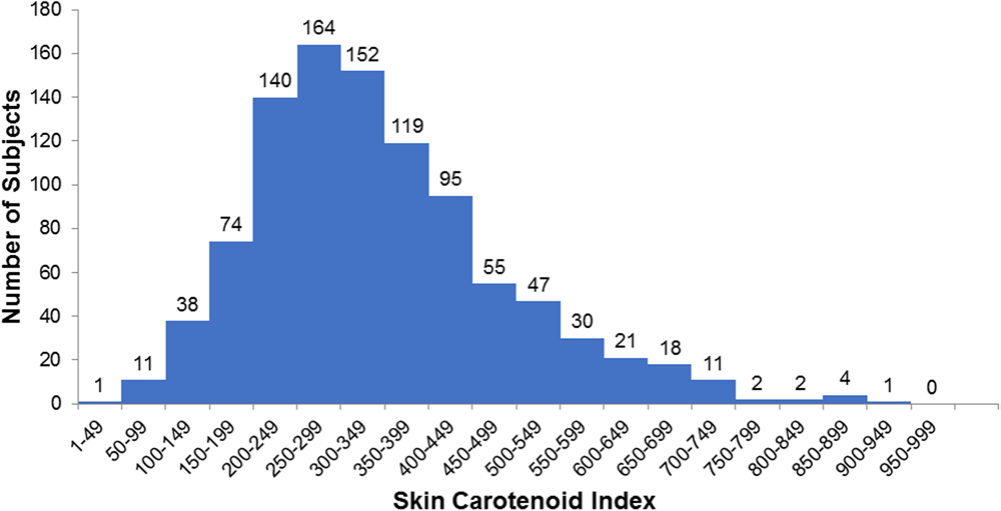
Histogram of skin carotenoid indices. The indices show a normal distribution with a slight skew to higher levels. The mean was 343.1 ± 142.1, (SD) and the median was 320 (95% CI, 334.2–352.0).

**Figure 3:**
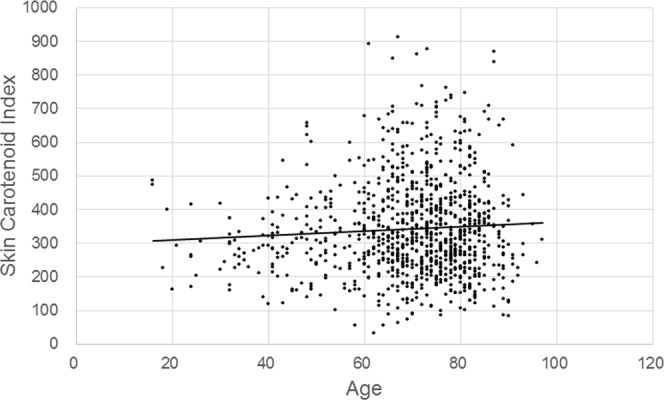
Skin carotenoid index and age in all subjects. The Pearson’s correlation coefficient was small but significant (r = 0.069, *P* = 0.035).

**Figure 4:**
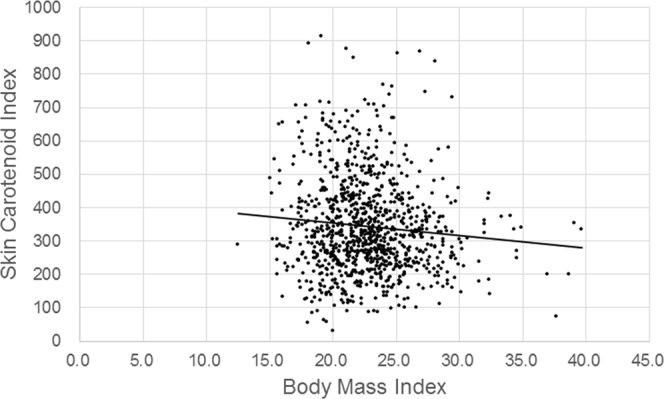
Skin carotenoid index and body mass index in all subjects. Pearson’s correlation coefficient was small but significant (r = −0.087, *P* = 0.008).

**Figure 5:**
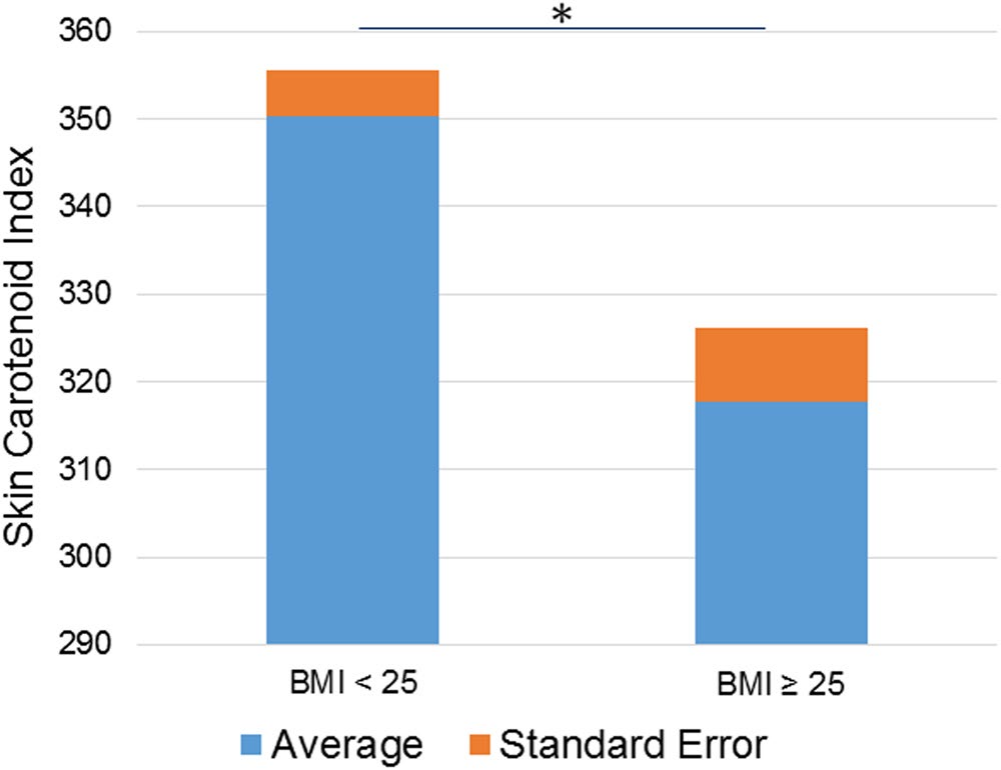
The mean skin carotenoid index of two groups classified by body mass index (BMI). Skin carotenoid indices of high BMI group was significantly lower than those of low BMI group (*P* < 0.001, t-test).

The SC indices of all subjects ranged from 32 to 914, and the mean was 343.1 ± 142.1 (SD). The corresponding histogram is shown in Fig. 2. Relationships between SC indices and age or BMI are respectively shown in Figs 3 and 4. The correlation coefficient for SC indices and age was 0.065 (*P* = 0.040, Pearson’s correlation coefficient test), and for SC indices and BMI it was −0.092 (*P* = 0.004, Pearson’s correlation coefficient test). When BMI was classified into two groups, with one containing normal and low body weight (BMI < 25, N = 766) subjects and the other one containing overweight subjects (BMI ≥ 25, N = 219), SC indices for overweight subjects (317.7 ± 126.5 (SD)) turned out significantly lower than normal and low body weight subjects (350.3 ± 145.5 (SD), *P* < 0.001, t-test) (Fig. 5).

The ranges and means of SC indices for the main patient groups are shown in Table 2. Women had significantly higher SC indices than men (*P* < 0.001, Welch’s t-test). Subjects supplementing with lutein had significantly higher SC indices than non-supplementing subjects (*P* < 0.001, Welch’s t-test). Significant differences in SC indices existed between the three smoking habit categories (*P* < t0.001, ANOVA), and multiple comparisons confirmed a significant difference between each pair of habits (*P* ≤ 0.001, Bonferroni). No statistically significant differences in SC indices existed between healthy subjects and subjects suffering from diabetes, or respectively, from any one of a large range of eye diseases, including AMD, high myopia, central serous chorioretinopathy, epiretinal membrane, macular hole, macular telangiectasia, and diabetic maculopathy.

**Table 2:** Skin carotenoid index of subjects with different characteristics.

		minimum	maximum	Mean (SD)	*P*
Sex	Men	32	869	315.4 (129.6)	**<0.001, (Welch’s t-test)**
	Women	117	914	382.1 (149.8)	
Lutein supplement	No	32	892	324.9 (130.2)	**<0.001, (Welch’s t-test)**
	Yes	136	914	414.9 (164.2)	
Smoking habit	No	57	914	372.5 (149.1)	**<0.001, (ANOVA)** **No/Past, <0.001** **No/Current, <0.001** **Past/Current, 0.001** **(Bonferroni)**
	Past	56	869	323.4 (124.5)	
	Current	32	863	272.9 (121.6)	
Diabetes	No	56	914	346.0 (142.5)	0.176 (t-test)
	Yes	32	723	330.3 (139.8)	
Macular disease	No	32	740	340.5 (141.9)	0.270 (ANOVA)
	AMD	56	914	346.2 (146.4)	
	Others	57	892	346.1 (135.9)	

The analysis for potential correlations between SC indices and all recorded blood test variables is summarized in Table 3. Alb and TC were significantly positively correlated, and Hb was negatively correlated, although the correlation coefficients were small. HbA1c had a marginal negative correlation.

**Table 3:** The correlation of skin carotenoid index and blood test variables.

	TP (410)	Alb (386)	Hb (416)	Cr (414)	eGFR (412)	TC (389)	TG (389)	LDL (68)	HDL (75)	LDL/HDL (58)	HbA1c (166)
r	0.034	**0.141**	**−0.105**	−0.093	0.022	**0.136**	0.007	0.028	0.132	−0.095	−0.148
*P*	0.490	**0.005**	**0.032**	0.060	0.655	**0.007**	0.889	0.819	0.257	0.480	0.056

(), number of examinations; r, Pearson’s correlation coefficient; TP, total protein; ALB, albumin; Hb, hemoglobin; Cr, creatinine; eGFR, estimated glomerular filtration rate; TC, total cholesterol; TG, triglycerides; LDL, low-density lipoprotein, HDL, high-density lipoprotein, HbA1c, hemoglobin A1c.

### Dependent and independent variables in multiple regression analysis

SC indices were chosen as dependent variables in the multiple regression analysis. All factors having significant influence on SC indices were chosen as independent variables. These included sex, age, lutein supplementation habit, smoking habit, BMI, Alb, Hb, and TC. Since subjects with AMD supplemented more frequently with lutein than others, macular disease was also included as an independent variable.

The results of all analyses are shown in Table 4. The coefficient of determination was 0.123. Tobacco smoking, lutein supplementation, and sex turned out as significant dependent variables.

**Table 4:** Multiple regression analyses of skin carotenoid indices.

	Partial regression coefficient	Standardized partial regression coefficient	*P*	95% confidential interval	
				Lower limit	Upper limit
Constant	377.4			357.5	397.4
Tobacco history	−46.6	−0.24	**<0.001**	−68.0	−25.2
Lutein supplement	54.0	0.13	**0.011**	12.5	95.4
Sex	−40.0	−0.14	**0.012**	−70.7	−8.6

*R*^2^ = 0.123, *P* < 0.001 (ANOVA).

### Skin carotenoid (SC) index and macular pigment (MP) optical density

A total of 150 eyes from 150 subjects with a healthy retina in at least one eye were included for further correlation analyses. When both subject eyes were healthy, the eye scheduled for cataract surgery at a later stage was chosen for analysis. The age of the subjects ranged from 18 to 93 years, with a mean of 72.5 ± 10.5 years; 60 were men, and 90 were women. All eyes but one had a visual acuity higher than 20/25. The exception was an eye with a visual acuity of 20/30.

Table 5 lists the mean values of the measured local MPOD levels at each retinal eccentricity, the MPOD volumes for the corresponding areas delineated by each eccentricity, and the correlation parameters between these MPOD volumes and SC indices. SC indices correlated significantly with local MPOD levels in the 0.43° to 4.02° eccentricity range, except for 1.99°, and they also correlated with the corresponding MPOD volumes. Correlation coefficients for 0° and 0.23° eccentricity were marginal.

**Table 5:** Local macular pigment optical density (MPOD) at nine eccentricities, MPOD volume within five eccentricities, and correlation with skin carotenoid indices.

Eccentricity	All subjects (150)			Subjects without lutein supplement (139)		
	Local MPOD (SD)	r	*P*	Local MPOD (SD)	r	*P*
0°	0.78 (0.20)	0.150	0.067	0.78 (0.18)	0.189	**0.026**
0.23°	0.76 (0.18)	0.156	0.060	0.76 (0.18)	0.184	**0.032**
0.43°	0.71 (0.18)	0.220	**0.008**	0.71 (0.18)	0.232	**0.007**
0.47°	0.71 (0.18)	0.231	**0.006**	0.71 (0.18)	0.239	**0.006**
0.51°	0.71 (0.18)	0.226	**0.006**	0.71 (0.18)	0.22	**0.008**
0.9°	0.70 (0.17)	0.203	**0.015**	0.70 (0.17)	0.172	**0.048**
0.98°	0.69 (0.17)	0.185	**0.025**	0.68 (0.17)	0.153	0.075
1.99°	0.35 (0.13)	0.135	0.105	0.35 (0.13)	0.103	0.233
3.01°	0.25 (0.09)	0.224	**0.007**	0.25 (0.09	0.190	**0.029**
4.02°	0.20 (0.07)	0.231	**0.005**	0.20 (0.07)	0.208	**0.016**
**Boundary**	**MPOD volume**	**r**	***P***	**MPOD volume**	**r**	***P***
0.47°	361.6 (84.8)	0.206	**0.013**	362.2(85.4)	0.224	**0.010**
0.9°	1248.4 (288.6)	0.231	**0.005**	1247.1 (293.0)	0.219	**0.011**
0.98°	1474.3 (349.8)	0.221	**0.007**	1472.4 (355.3)	0.209	**0.014**
1.99°	4487.1 (1232.9)	0.154	0.061	4469.0 (1257.1)	0.124	0.146
3.01°	7423.6 (2278.5)	0.186	**0.026**	7388.4 (2324.6)	0.154	0.077
4.02°	10655.7 (3412.0)	0.188	**0.024**	10597.9 (347509)	0.154	0.078
8.98°	20187.2 (6672.1)	0.223	**0.006**	20079.6 (6748.7)	0.12	**0.024**

It is well-known that lutein supplementation effectively induces an increase in MP levels/volumes, but whether lutein supplementation leads to increased SC levels is still unknown. Therefore, we analyzed the correlation between MPOD levels and SC indices in 139 subjects not supplementing with lutein, and the results were similar.

## Discussion

The mean SC index for the 985 subjects was 343.1 ± 142.1 (SD), with lower indices in smokers and men relative to non-smokers and women. Subjects supplementing their diet with lutein scored higher than non-supplementing subjects. SC indices correlated significantly with MPOD levels, albeit with a rather modest coefficient.

The histogram of SC indices, ranging from a lowest index of 32 to a highest index of 913, shows that large differences (up to ~30-fold) can exist between subjects for their diet-derived skin carotenoid concentrations. The envelope of the histogram is near normal, with only a slight skew towards higher indices. These features are very similar to a previously published distribution pattern for a large population group of 32,942 subjects screened for SC levels in the U.S. via RRS^31^. Those RRS based SC levels ranged from 2,000 to 78,000, had a mean of 24,000. The minor difference in widths can be attributed to a combination of slightly different sensitivities between the two methods (a bit higher for RRS), numbers of the respective subject populations (1000 for RS vs 33,000 for RRS), and differences in the numbers of devices used for the data collection (one of RS, multiple devices for RRS).

It is interesting to compare our screening results with emerging data for other population groups. In a study involving SC screening of 54 healthy participants in a U.S. eye clinic via RS, the mean SC index was 297^11^. In a screening study involving customers of a convenience store in Eastern North Carolina, the mean SC indices obtained via RS were 239 ± 85.8 (SD) for African Americans and 225.4 ± 87.8 (SD) for Non-African Americans^32^. In comparison with these groups, the mean SC index for our Japanese population is significantly higher. As we did not examine the dietary habits of the screened participants, we cannot conclude whether this difference is due to dietary habits or effects on the underlying absorption of carotenoids into the tissue.

SC indices were 21% higher in women than men, 27% higher in non-smokers than current smokers, and 32% higher in subjects taking lutein supplements. Also, there is no age effect. These results were similar to those in previous studies^11,33–35^, independent of the fact that most of the previous studies had used RRS for the skin measurements.

In human skin, lycopene and beta-carotene are predominant, while lutein accounts only for a much smaller percentage. Scarmo *et al*.^36^ reported lycopene and beta-carotene account for 76% of total carotenoids and lutein accounts for only 1.5% in punch-biopsied abdominal skin samples that contained also a relatively large fraction of subcutaneous tissue. But in much thinner, heel skin samples, Ermakov *et al*.^8^ recently reported that lutein accounts for ~15% of total carotenoids. Increases.of SC levels upon beta-carotene, lycopene, and vegetable juice consumption have been reported previously^11,34,35,37^ however, no interventional study has been performed yet with lutein as the sole carotenoid species. Since most Japanese lutein supplements contain only lutein or lutein and zeaxanthin but no other accompanying carotenoids, our finding can be viewed as indirect evidence that lutein also leads to increased SC levels, which would be consistent with their optical absorption in the same spectral region.

AMD is a multifactorial disease. Oxidative stress caused by blue light and tobacco smoking has been established as one of the major causes. Subjects with high SC indices likely have high antioxidant activity in their light exposed tissues, which would include the retina. Therefore, we had speculated that subjects with high SC levels may have a lower frequency of AMD than subjects with low SC levels. Looking at the respective SC levels of the subjects without lutein supplementation, the mean SC index for AMD cases was 316.2 ± 127.6, while the index for healthy subjects was 331.1 ± 131.7, which is only an insignificant difference (*P* = 0.116, t-test). The low correlation coefficient between SC indices and MPOD levels can be considered as a major reason for this result.

In the multiple regression analysis, the absolute value of the standardized partial regression coefficient for tobacco smoking was higher than the values for gender and lutein supplementation. Smoking therefore is a stronger factor than gender for reduced SC indices, and lutein supplementation should be recommended particularly for male smokers. Obviously, the low decision factor of 0.123 of the multiple regression analyses clearly points to powerful additional factors affecting SC indices. Among these, dietary habits and genetic background can be assumed to be most important. Future studies designed to include these factors are needed to quantify their respective contributions.

Since 42% of all subjects underwent blood tests, we looked at correlations between SC indices and various blood analytes. Alb and TC levels revealed a positive correlation, albeit with small correlation coefficients. Since Alb levels in the blood are related to general nutrition, subjects with malnutrition or poor nutrient absorption can be expected to also have lower SC indices. Hb was negatively correlated with SC indices, but again with a very low correlation coefficient. Since blood is temporarily pushed out from the measured tissue volume by the Veggie Meter’s finger cover pressure, effects of residual tissue blood levels on the SC measurements can be excluded. The obtained result may warrant future studies into potential relationships between SC levels and biomarkers in blood.

The present results show a marginal, yet statistically significant correlation between SC indices and local MPOD levels and associated MPOD volumes for all retinal eccentricities investigated (with only one exception for 1.99°, see Table 5). The results were independent of lutein supplementation. These relatively low correlations are at variance with previous published higher correlations^20–22^. Conrady *et al*.^22^ reported a coefficient of 0.663 between SC indices and total MPOD volume within 8.98°, while our results revealed only low coefficients of 0.231 for volume and local MPOD correlations. The main differences between their study and ours is the device used for the measurement of SC levels, the subject race, age, and the lens transparency. The methodology for the skin measurement can be ruled out as a major factor in view of the high correlation between RRS and RS (the squared correlation coefficient (R^2^) is 0.85^29^ or 0.88^11^), leaving differences in subject age (59 ± 17 years in the previously published study^22^ versus 72.5 ± 10.5 years in the present one), race, and the lens status as likely reasons for the lower correlation outcome, but further studies with larger sets of MPOD data will be needed for clarification.

The shortcomings of the present study were as follows. Most subjects were ophthalmology clinic patients, and the number of healthy volunteers was relatively small. Therefore, further screening results are needed for healthy subjects outside the hospital environment. The age range of subjects was large (16 to 97 years), but elderly subjects were in the majority. Further studies need to include also younger subjects. Serum carotenoid levels were not measured, and dietary habits and genetic background were not evaluated. Lutein supplementation was self-reported, and important factors such as supplement ingredients and supplementation duration, were not available.

A strength of this study is the large population of almost 1000 subjects participating in the SC measurements, allowing us to look into the presence or absence of correlations between SC levels and numerous health biomarkers with high statistical significance. Also, MPOD was measured only in pseudophakic eyes, limiting the potential influence of cataract^38^. MPOD levels were analyzed not only at discrete eccentricities but also for the related, more integrative MPOD volumes.

SC indices were measured easily and safely using RS, and the present data can be used as an initial reference set for Japanese subjects.

## Methods

### Subjects

A total of 985 subjects were included in this study: 957 were patients at the Department of Ophthalmology of Seirei Hamamatsu General Hospital, and 28 subjects were healthy volunteers (Department of Ophthalmology staff). Subjects were 16 to 97 years old, with a mean age of 69.7 ± 13.6 (standard deviation, SD) years. Men accounted for 577 subjects, and women for 408. SC levels were measured in all subjects, and the MPOD levels were measured in 191 subjects. The institutional review board of Seirei Hamamatsu General Hospital approved this prospective case series (IRB No. 2189, 2251, 2253). The protocol followed the tenets of the Declaration of Helsinki. All patients and a parent and/or legal guardian of the subjects under the age of 18 years provided written informed consent.

### Measurement of skin carotenoid levels

We used pressure-mediated RS (Veggie Meter, Longevity Link Corporation, Salt Lake City, Utah) to measure SC levels. The basics of this device have been described elsewhere^29^. Measurements were performed following the instructions of the device manufacturer. Calibration was performed with the provided dark and white reference materials prior to daily skin measurements. Subjects inserted the left middle finger into the device’s finger cradle and had the tip pushed against the convex contact lens surface with the help of a spring-loaded lid. The modest pressure applied to the fingertip reduced the blood perfusion of the measured tissue volume, in this way preventing the strongly absorbing blood from interfering with the measurement of skin carotenoid levels. The SC index was determined as the average of three consecutive measurements.

Measurements were performed after ophthalmological examinations on the same day. All subjects underwent visual acuity testing, measurement of intraocular pressure, fundus examination by ophthalmoscope, fundus photography with mydriasis induced using 2.5% phenylephrine hydrochloride and 1% tropicamide. Additional examinations such as optical coherence tomography and fluorescein angiography were performed as needed. Height and body weight were measured as well. The subjects were asked whether or not they were taking supplements containing lutein. Details about exact ingredients or supplementation duration and dosage were not recorded, but all subjects reported taking supplements for at least one month.

### Measurement of macular pigment optical density

We used a prototype MPOD module installed on a Heidelberg Spectralis^®^ MultiColor platform (“Spectralis-MP”, Heidelberg Engineering, Heidelberg, Germany). This device used 486 nm and 517 nm excitation wavelengths. The basic functionality and handling of this instrument is described elsewhere^39,40^. The beam diameter at the subject’s pupil was 3 mm; autofluorescence images of the 30° central area of the retina were recorded for both wavelengths with pupil dilation. The optical densities at ten eccentricities, i.e., 0°, 0.23°, 0.43°, 0.47°, 0.51°, 0.9°, 0.98°, 1.99°, 3.01°, and 4.02° eccentricities (local MPODs), and the optical density volume within seven eccentricities, i.e., 0.47°, 0.9°, 0.98°, 1.99°, 3.01°, 4.02°, and 8.98° (MPOD volume), were used for analyses. The MP was assumed to be negligible at 8.98° eccentricity, and this eccentricity was chosen as zero background level. The local MPOD levels are averages along concentric circles at selected eccentricities except for 0°. Since the method is affected by cataracts^38^, we limited measurements to the 191 subjects who had already undergone cataract surgery. Measurement was performed on day 3 or 4 after cataract surgery, following the above-mentioned ophthalmological exams and measurements of SC indices.

### Blood tests

The study protocol did not include lutein or zeaxanthin analysis in plasma nor other blood tests. However, many subjects had already undergone blood tests for various medical conditions. We reviewed all those blood tests from medical records that were carried out within one month of measuring the SC levels. Blood tests were performed in 417 subjects; 165 subjects underwent testing for general condition checkup before cataract surgery, and 252 subjects underwent testing for systemic diseases while being treated by other hospital departments. Since the purpose of the blood test was different for each subject, the test items varied. We chose common items for all tested subjects, i.e., total protein (TP), albumin (Alb), hemoglobin (Hb), total cholesterol (TC), triglycerides (TG), low-density lipoprotein (LDL), and high-density lipoprotein (HDL) to evaluate general condition. Creatinine (Cr) and estimated glomerular filtration rate (eGFR) were chosen to evaluate renal function, and hemoglobin A1c (HbA1c) was chosen to evaluate blood sugar control.

### Statistical analyses

The comparison of two average values of numerical variables was performed using t-tests. Categorical data were analyzed using a chi-square test. The correlations between two numerical variables were investigated using Pearson’s correlation coefficient test. The comparison of SC indices between the three smoking habit categories was performed using one-way analysis of variance (ANOVA) with Bonferroni multiple comparisons. Multiple regression analysis was performed with SC indices as dependent variables, and factors that influenced SC indices were chosen as independent variables using a stepwise method. Statistical analyses were performed using IBM SPSS Statistics 25 software, and a value of *P* < 0.05 was considered statistically significant.

## Data Availability

The datasets generated during and/or analyzed during the current study are available from the corresponding author on reasonable request.
